# Measuring women’s experiences during antenatal care (ANC): scoping review of measurement tools

**DOI:** 10.1186/s12978-023-01653-5

**Published:** 2023-10-10

**Authors:** Hedieh Mehrtash, Karin Stein, Maria Barreix, Mercedes Bonet, Meghan A. Bohren, Özge Tunçalp

**Affiliations:** 1 UNDP/UNFPA/UNICEF/WHO/World Bank Special Programme of Research Development and Research Training in Human Reproduction (HRP), Department of Sexual and Reproductive Health and Research, World Health Organization (WHO), Geneva, Switzerland; 2 Department of Global Health, University of Washington School of Public Health, Seattle, Washington USA; 3 Gender and Women’s Health Unit, Nossal Institute for Global Health, University of Melbourne, Melbourne, Australia

**Keywords:** Antenatal care, Women's experiences of care, Respectful maternity care, Measurement

## Abstract

**Background:**

The new WHO model for antenatal care (ANC) focuses on improving practice, organisation and delivery of ANC within health systems, which includes both clinical care and women’s experiences of care. The goal of this review is to identify tools and measures on women’s experiences of ANC.

**Methods:**

We conducted a scoping review to identify tools and measures on women’s experiences of ANC. An iterative approach was used to review all tools in a series of four steps: (1) identify papers between 2007 and 2023; (2) identify the tools from these papers; (3) map relevant measures to conceptualizations of experiences of care, notably mistreatment of women and respectful maternity care and (4) identify gaps and opportunities to improve measures.

**Results:**

Across the 36 tools identified, a total of 591 measures were identified. Of these, 292/591 (49.4%) measures were included and mapped to the typology of mistreatment of women used as a definition for women’s experiences care during ANC in this review, while 299/591 (44.9%) irrelevant measures were excluded. Across the included measures, the highest concentration was across the domains of poor rapport between women and providers (49.8%) followed by failure to meet professional standards of care (23.3%). Approximately, 13.9% of measures were around overall respectful care, followed by health systems (6.3%), and any  physical or verbal abuse, stigma and/or discrimination (4.8%) .

**Conclusion:**

This analysis provides an overview of the existing tools, gaps and opportunities to measure women’s experiences during ANC. Expanding beyond the childbirth period, these findings can be used to inform existing and future tools for research and monitoring measuring women’s experiences of ANC.

**Supplementary Information:**

The online version contains supplementary material available at 10.1186/s12978-023-01653-5.

## Background

With a growing recognition that coverage of care alone is insufficient in improving health, efforts to improve maternal health are increasingly focused on increasing the quality of care, including both provision and experience of care, critical components of Universal Health Coverage (UHC) [1, 2]. Experience of care refers to the interpersonal aspects of the quality of care provided. Experiences of care are considered ‘process indicators,’ and the reference considered is user’s self-reported experiences with the health system [3]. For women, improving experiences of care across the continuum of maternal and newborn care is imperative, as it is a fundamental right which can produce better health outcomes and health care utilization [4].

Experience of care in this context includes but is not limited to effective communication, respect and dignity, and access to the social and emotional support of her choice [1, 5]. There has been substantial progress in developing tools and methods to measure this phenomenon [6, 7]. A global scoping review by Larson and colleagues assessed tools measuring women’s experiences across the continuum of maternal and newborn care, from antenatal care (ANC) through postnatal care (PNC) [6]. The paper found a diverse set of measures and measurement tools/instruments currently used to assess each component of the care continuum; however, most (80%) existing tools focused on care during childbirth (intrapartum care). Understanding and applying what is known about measuring experiences of care during the childbirth period to the comparatively understudied ANC and PNC periods is a critical gap.

ANC is usually the first interaction between a pregnant woman and the health system, and poor experiences of ANC may influence her future choice of facility or health worker, or in absence of options, ultimately influencing her decision on seeking facility-based childbirth care entirely [8]. The 2016 WHO recommendations on ANC for a positive pregnancy experience present a woman-centered approach to ANC, by providing evidence-based interventions focusing on the quality of care [9, 10]. With this new model which includes eight ANC contacts with health workers for routine care, WHO also developed a monitoring framework which includes an emphasis on women’s experiences of ANC services for a positive pregnancy [10].

The aim of this analysis is to conduct a scoping review [6] with a focus on tools and measures that capture women’s experiences of ANC and to identify critical gaps and opportunities for future research and implementation.

## Methods

**Fig. 1:**
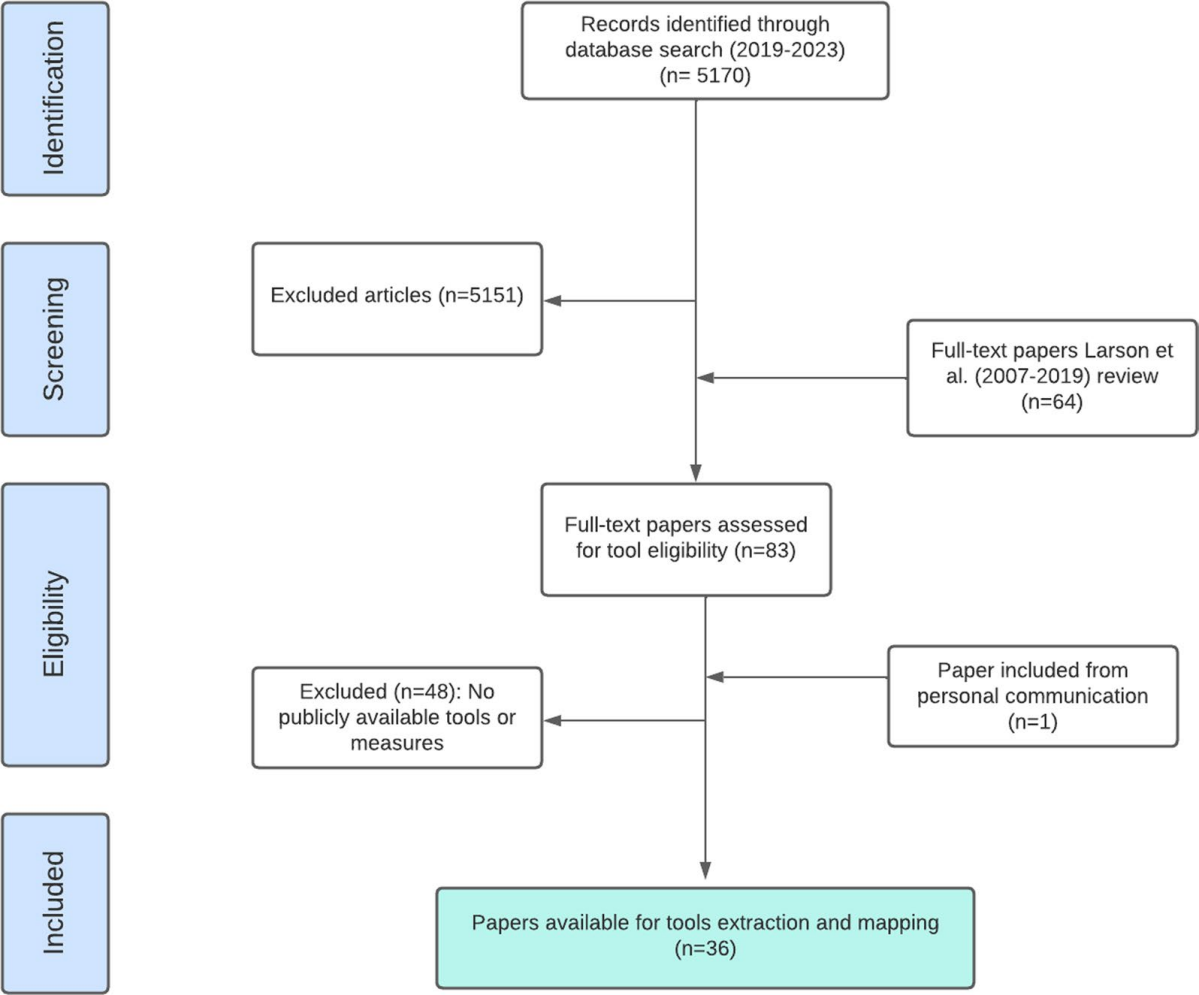
PRISMA flow diagram

**Fig. 2:**
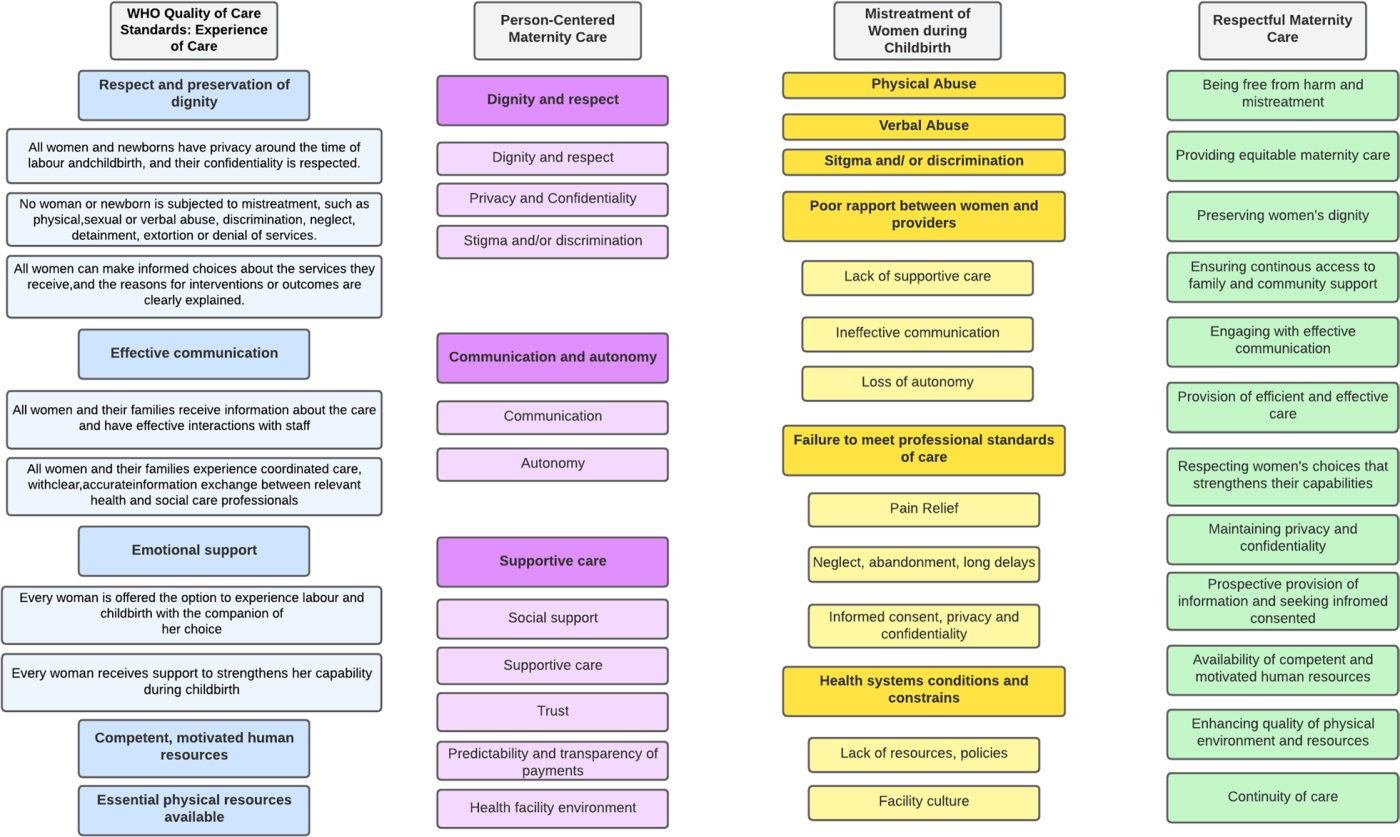
Overview of experience of care conceptualizations^1^. ^1^Dark and Light Blue: WHO Experience of Care Framework [1, 5]; Dark Pink: Domains of Person-Centered Maternity Care, Light Pink: Sub-domains of Person-Centered Maternity Care [13]; Dark Yellow: Domains of Mistreatment of Women, Light Yellow: Sub-Domains of Mistreatment of Women [11]; Green: Domains of respectful maternity care [12]

An iterative approach was used to review all tools and measures in a series of four steps : (1) identify studies published between 2007 and 2023 (Fig. 1); (2) identify the full tools and measures from the identified studies in step 1 ; (3) map relevant measures to conceptualizations of experience of care, notably mistreatment of women and respectful maternity care [11, 12] and; (4) identify gaps and opportunities for future measures.We used two primary sources to conduct the scoping review for papers on experience of care during ANC between 2007 and 2023.  

### Step 1: Identify papers between 2007 and 2023

The first source to identify papers was the scoping review from Larson et al. [6] for the period between January 2007 to February 2019 [6]. Briefly, the authors presented a scoping review of published literature to identify measures and tools related to women’s experiences of facility-based care across the continuum of maternity care including antenatal, intrapartum, postnatal care, and abortion using bibliographic databases (PubMed, Embase, CINAHL, Web of Science and Global Index Medicus). Studies were eligible for inclusion in the Larson et al. review if they were original research (i.e., not an editorial, comment or newspaper article), study participants were women who are/were pregnant, study reported on facility-based care for pregnant or postpartum women with newborns and results included those from a quantitative research study of any design. We identified and included papers on ANC that were identified from the period this review was conducted (2007–2019).

Our second source included conducting an updated search for additional papers on women’s experiences during ANC published between February 2019 and May 2023 using both search strategies from Larson et al.[6] and Downe et al. [8] (Additional file 1: Annex I). One additional relevant paper (and related tool) from unpublished literature was identified from personal communication and included.

### Step 2: Identify full tools and measures from papers

Based on the papers identified during step 1, the next step was to extract available data collection tools (e.g. questionnaires, surveys) and/or measures (e.g. questions, survey items) used to evaluate women’s experiences of ANC. These were then grouped into four categories: studies that used (i) a validated tool, (ii) some components of a validated tool, (iii) non-validated tools and (iv) validation studies. All measures from the tools were documented in a spreadsheet and papers without publicly available tools (e.g., tools that were cited but could not be found in other peer-reviewed publications, supplementary appendices, nor external website) were subsequently excluded. Three authors (HM, OT, MB) reviewed the papers to identify publicly available tools.

### Step 3: Mapping of identified measures to conceptualizations of experiences of care

The WHO defines experience of care for pregnant women along three components: (1) effective communication; (2) respect and dignity; and [3] emotional support [1, 5]. Various terms (for example, “respectful maternity care,” “culturally-appropriate care,” “obstetric violence,” “dehumanized care,” “disrespect and abuse”, “mistreatment of women”, “person-centered maternity care”) have been used to describe this phenomenon during childbirth [13–16]. We provide an overview of the most widely used conceptualizations of experiences of care developed mainly for intrapartum care to assess similarities, differences and potential application to ANC. The conceptualizations include: (i) WHO quality of care framework (experience of care), (ii) person-centered maternity care, (iii) mistreatment of women during childbirth and (iv) respectful maternity care (Fig. 2) [1, 5, 11–13]. The assessment illustrated that these conceptualizations are highly inter-related. For the purposes of this paper, the mistreatment of women domains were utilized as they were more specific compared to the other conceptualizations (Additional file 1: Annex II—typology of mistreatment of women during childbirth, Fig. 2—overview of conceptualizations of experience of care).

ANC measures identified through the review of tools in Step 2 were mapped to the domains of the mistreatment of women typology. These are highlighted in dark yellow in Fig. 1: physical abuse, verbal abuse, stigma and/or discrimination, failure to meet professional standards, poor rapport with healthcare providers, and health system conditions and constraints [11]. In addition, we categorized overall respectful care measures (such as receipt of respectful dignified care, treated with respect and being free from harm and mistreatment). During the mapping process, no modifications were made to the existing measures identified from the review of tools (Additional file 1:  Annex III—list of tools and measures).

It is important to note, however, that there might be certain areas of experience of ANC that are not covered by the conceptualizations that were original developed for intrapartum care. Therefore, any additional ANC-specific areas that were identified as part of this study’s empirical work were not only categorized into an existing domain and sub-domain but also as an emerging area for further exploration and research.

One reviewer categorized (HM) the included measures to the domain and, a second reviewer (KS) evaluated the categorization process. Lastly, a third reviewer (ÖT) addressed discrepancies through discussion with the two reviewers, until consensus was reached.

### Step 4: review available measurement tools and measures for gaps and opportunities

Descriptive univariate analysis was used to produce summary statistics (frequencies and percentages) of the measures from Step 3 (identified measures from Step 2 that were mapped to existing domains). Gaps and opportunities for novel measures for women’s experiences of ANC were identified, based on the mapping process and classified as emerging subitems.

## Results

A total of 36 tools from 2007 to 2023 were identified for the next stage of the framework mapping process. The majority (22/39, 57.9%) were from high-income countries (Australia, Canada, France, Hungary, India, Italy, Netherlands, Norway, Qatar, Singapore, United Kingdom, USA). 43.6% (17/39) were from low, lower-middle, or upper-middle income countries (Benin, Brazil, Ethiopia, India, Kosovo, Malawi, Nepal, Nigeria, Pakistan, Rwanda, South Africa). Lastly, an additional unpublished tool from a study in India was received, reviewed and included [17].

Across the 39 tools identified, a total of 591 measures were identified. Of these, 292/591 (49.4%) measures were included and mapped to the typology of mistreatment of women used as a definition for women’s experiences care during ANC in this review, while 299/591 (44.9%) irrelevant measures (e.g. around provision of clinical care) were excluded.

292 measures relevant to women’s experiences during ANC were classified across the five domains of the mistreatment typology (Additional file 1: Annex III – list of tools and measures). The highest frequency of measures observed are across the *poor rapport between women and providers* domain [18–32] (143/292 measures, 49.0%), followed by *failure to meet professional standards of care* domain [20, 21, 25, 27–37] (67/292 measures, 22.9%). The lowest frequency of measures was across *any physical* [33, 35], *verbal abuse* [21, 33], *stigma and/or discrimination* [20, 25, 33] (14/292 measures, 4.8%) and the *health systems conditions and constraints* domain [21, 28, 31, 33, 34, 38] (18/292 measures, 6.3%).

Additionally, overall respectful care measures comprised approximately 15% (40/292, 13.9%) (e.g., “*Were you treated with respect?”, “Did you receive respectful care”, “were you treated with respect and dignity?”)* and these were assigned to the being free from harm and mistreatment and preserving women’s dignities domains of respectful maternity care (Table 1).

**Table 1:** Measures related to ANC mapped to the mistreatment typology domains and subdomains

Domain and subdomains	Measures for antenatal care mapped across domain and sub-domain (N = 292) N (%)
Total measures across all	292
**Any physical abuse, verbal abuse, stigma and/or discrimination**	14 (4.8)
Physical abuse	3 (21.4)
Verbal Abuse	5 (35.7)
Stigma and/or discrimination	6 (42.9)
**Failure to meet professional standards of care**	67 (23.3)
Informed consent and confidentiality	35 (52.2)
Neglect, abandonment, and long delays	31 (46.3)
Pain relief	1 (1.5)
**Poor rapport between women and providers**	143 (49.8)
Ineffective communication	69 (48.3)
Loss of autonomy	28 (19.6)
Lack of supportive care	46 (32.2)
**Health systems conditions and constraints**	18 (6.3)
Lack of resources	15 (83.3)
Facility culture	3 (16.7)
**Overall respectful care**	40 (13.9)
**Other (e.g. mental health, etc.)**	9 (3.1)

While we were able to categorize most measures across the mistreatment of women typology, we found that there were certain areas pertaining to ANC where the typology was limited and would need to be expanded. Table 2 describes the sub-items within the *poor rapport between women and providers* and *health systems conditions constraints* that could potentially be new measures and need additional empirical work for inclusion in an adapted version of the mistreatment of women typology for ANC. Within the *poor rapport between women and providers* domain, 18/76 (23.6%) measures were related to provision of information about care, 5/46 (10.8%) to provision of empathetic care and, 6/28 (21.4%) to decisions taken about care. Within *health systems conditions constraints* domain, 8/15 (53.3%) measures were mostly related to enabling environment.

**Table 2:** Gaps and opportunities for potentially new measures within mistreatment typology domains relevant to ANC

Original sub-item	Emerging sub-item (categorized within the original sub-item)
**Poor rapport between women and providers**	
Ineffective communication (n = 76)	Provision of information related to care (n = 18) (e.g., were you told where to go if you had any pregnancy complications; provides information about care; provides information about prenatal tests)
Lack of supportive care (n = 46)	Empathetic care (n = 5) (e.g., did the provider show that they cared about you; was care provided in an empathetic manner)
Loss of autonomy (n = 28)	Decision about care (n = 6) (e.g., do you completely trust the health worker’s decisions about medical treatments in this facility)
**Health systems conditions and constraints**	
Lack of resources(n = 15)	Enabling environment (n = 8) (e.g., availability of chairs, clean toilets, and rooms)

## Discussion

This scoping review aimed to provide an overview of the current state of tools and measures used in research on women’s experience of facility-based ANC and mapped these tools to an existing typology [11, 12]. We identified 292 unique measures across various tools assessing experience of ANC with large variations of the number of measures per domain. Findings from previous analyses have suggested that delivery of high quality ANC should incorporate experience of care along with effective provision of clinical care [6, 8]. Based on the mistreatment of women typology, most findings from our review pertain to the domains of *poor rapport between women and providers*, and *failure to meet professional standards of care*. Despite growing evidence and focus on physical and verbal abuse during childbirth period, there were few measures identified on physical or verbal abuse in the context of ANC. Given what we know about the timing of physical and verbal abuse around the time of childbirth (e.g. increasing particularly in the 15–60 min before birth), it may be that physical and verbal abuse are inherently less likely to happen in the context of ANC; however, further research is needed to explore this phenomenon during ANC [39].

Across measures identified in our review, half pertained to the *poor rapport between women and providers* domain which relates to ineffective communication, autonomy, and supportive care. This is not surprising given what has emerged from the ANC literature on what matters to women for achieving a positive pregnancy experience, which highlights the importance of effective communication as well as emotional support, self-esteem and autonomy [8]. Within this domain, ineffective communication measures comprise half of the currently available and identified questions. An interesting finding was the variation of the type of ineffective communication measures in ANC compared to childbirth, which focuses more provision of information such as details of exams, tests, procedures, and prescriptions tests as well as communication between health worker and pregnant women. Similarly, autonomy regarding decisions about care was found as part of the measures that play a role in women’s autonomy during ANC, overall. A Cochrane qualitative synthesis evidence review has also documented the importance of provision of information as playing a crucial role in the uptake of ANC services which is inextricably linked to ensuring women have autonomy for decision-making during their pregnancies [40]. Further empirical and qualitative research efforts are needed to better understand how best to measure how women receive information in a manner and format that is understandable to them and are supported to make informed decisions during their ANC contacts.

In terms of the *failure to meet professional standards of care* domain, informed consent and confidentiality sub-domains have been documented across the tools and measures in this exercise. However, it is important to emphasize that measures on consent and counseling for examinations, tests, and procedures should be incorporated as part of the routine ANC services. Furthermore, it is imperative women are made aware of and understand the importance of informed consent for upcoming procedures (e.g., vaginal examinations) during childbirth such that mistreatment can be prevented. A study across three countries found that almost half of vaginal examinations were non-consented during childbirth [41].

Lastly, the growing evidence on respectful care is a result of various drivers, particularly *health systems’ constraints*, that must be addressed across the continuum of maternity care [4]. Our review found limited measures pertaining to health systems constraints; however, some examples included provision of a comfortable physical setting and availability of resources at the facility to support care. To provide high quality experience of ANC, we must be able to incorporate measurement around health systems to capture the availability of an enabling and empowering environment (e.g. clean toilets, curtains during examinations) and health workforce to support women during pregnancy which has been reinforced across all experience of care conceptualizations to date.

Another important and emerging area of poor experiences of care has been during the COVID-19 pandemic where the provision of quality maternal and newborn care was compromised. A global survey found that COVID-19 pandemic negatively affected provision of and experience of care due to health workers’ fear of getting infected and measures taken to minimize COVID-19 transmission [42]. The COVID-19 pandemic reversed and put efforts to advance delivery of better women’s experiences of care at risk. Moving forward, innovations and interventions that could promote high quality experience of care across the continuum of maternity care globally are needed.

There are several implications from our review that can be incorporated into existing and future tools developed for measuring women’s experiences of ANC. Experience of care during the ANC period has been identified as a priority area in the WHO monitoring framework, which aims to provide guidance to countries on how to monitor the implementation and impact of routine ANC [10]. As such, the evidence-based measures and domains identified in this review can help implement the new WHO ANC monitoring framework, by providing further knowledge to support the development and implementation of global measures on experience of ANC. Furthermore, the measures identified in this review will be used to inform the experience of ANC measures as part of future implementation research on the WHO ANC model, informing WHO’s work in this area [43]. Additionally, given that this is a priority area for WHO and the Human Reproduction Programme (HRP), the continuation of this work will be expanded across the continuum of maternity care beyond ANC to PNC, to capture women’s experiences in the postnatal period and to ensure women achieve a positive motherhood experience [44, 45]. Finally, across the continuum of maternity care, more work is needed to ensure that proposed quantitative measures are understandable and answerable by women across contexts, ensuring participant’s perceptions, expectations and experiences of care (e.g. more nuanced descriptions of kindness or empathy or mutual decision making) reflect socio-contextual issues embedded in various settings (e.g. fragile settings). For example, cognitive interviewing is a qualitative approach that assesses whether quantitative survey questions assess the intended cognitive domain among respondents [46], which could be employed to further refine items/questions on the missing, or less researched domains, before implementation. 

This analysis has several strengths and limitations. In terms of strengths, this is one of the few studies comprehensively evaluating existing experience of ANC measures, globally. Regarding limitations, firstly, the conceptualizations and typologies around experience of care (including the ones we used for the results) were originally developed for intrapartum care and might not cover all the specificities around ANC. Secondly, a lack of available tools among the studies published limited our ability to assess all measures. Thirdly, not all measures included in this review are validated (Additional file 1: Annex III—list of tools and measures) and require further testing.

## Conclusion

While efforts to measure women’s experiences of care have grown significantly in the past decade, gaps remain in assessing this phenomenon across the continuum of maternity care, including ANC. This exercise provides an overview of the existing tools, gaps and opportunities to measure women’s experiences of ANC. Careful testing and adaptation of questions/items to local contexts will be important to ensure these measures accurately capture pregnant women’s experiences.

### Supplementary Information

**Additional file 1. Annex I.** Search strategy. **Annex II. **Typology of the mistreatment of women during childbirth. **Annex III.** List of tools and measures available.

## Data Availability

Data are available upon request.
