# Supplementary material for: Measuring women’s experiences during antenatal care (ANC): scoping review of measurement tools
Source: Reprod Health. 2023 Oct 10;20:150. doi: 10.1186/s12978-023-01653-5 (PMC10565981; doi:10.1186/s12978-023-01653-5)
Supplement: Supplementary file 1 — Additional file 1. Annex I. Search strategy. Annex II. Typology of the mistreatment of women during childbirth. Annex III. List of tools and measures available. [file 12978_2023_1653_MOESM1_ESM.docx]

# Additional file 1

# Annex I Search Strategy

**Larson et al. 2015- January 2019**

(maternal health[tiab] OR maternal service*[tiab] OR maternity care[tiab] OR maternal care[tiab] OR maternity service*[tiab] OR "Maternal Health"[mesh] OR "Maternal Health Services"[mesh]) AND (experience[tiab] OR experiences[tiab] OR patient-centered[tiab] OR woman centered[tiab] OR women centered[tiab] OR client centered[tiab] OR satisfaction[tiab] OR social support*[tiab] OR emotional support*[tiab] OR provider choice[tiab] OR choice of provider[tiab] OR wait time*[tiab] OR affordability[tiab] OR dignity[tiab] OR respect[tiab] OR privacy[tiab] OR confidentiality[tiab] OR discrimination[tiab] OR communication[tiab] OR disrespect[tiab] OR abuse[tiab] OR mistreatment[tiab] OR perception*[tiab])

**Search Update February 2019- May 2023**

(antenatal or prenatal or antepartum [Ti or Ab]) ; AND (experience[tiab] OR experiences[tiab] OR patient-centered[tiab] OR woman centered[tiab] OR women centered[tiab] OR client centered[tiab] OR satisfaction[tiab] OR social support*[tiab] OR emotional support*[tiab] OR provider choice[tiab] OR choice of provider[tiab] OR wait time*[tiab] OR affordability[tiab] OR dignity[tiab] OR respect[tiab] OR privacy[tiab] OR confidentiality[tiab] OR discrimination[tiab] OR communication[tiab] OR disrespect[tiab] OR abuse[tiab] OR mistreatment[tiab] OR perception*[tiab])

# Annex II – Table 1. Typology of the mistreatment of women during childbirth.


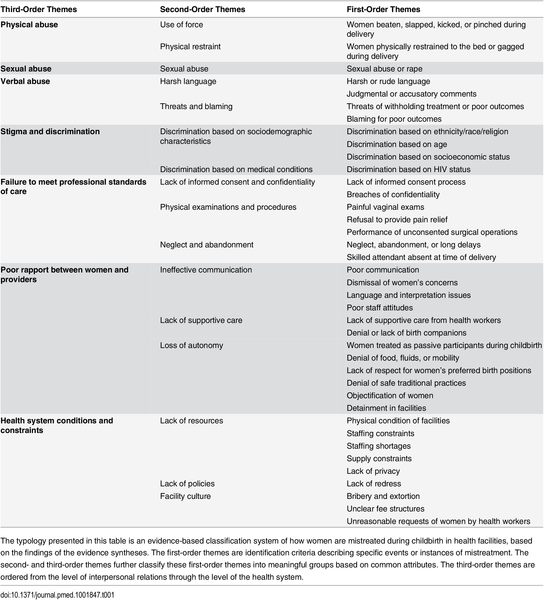


Bohren MA, Vogel JP, Hunter EC, Lutsiv O, Makh SK, et al. (2015) The Mistreatment of Women during Childbirth in Health Facilities Globally: A Mixed-Methods Systematic Review. PLOS Medicine 12(6): e1001847. https://doi.org/10.1371/journal.pmed.1001847

<https://journals.plos.org/plosmedicine/article?id=10.1371/journal.pmed.1001847>

# Annex III – List of tools and measures available

Please visit for a complete list of tools and measures: <https://docs.google.com/spreadsheets/d/1CuWdOinK7Fhf8dLCMyGeQiRdA0K9vldRUrCVKEBZ6Kg/edit?usp=sharing>
